# Assessing opportunities and inequities in undergraduate ecological forecasting education

**DOI:** 10.1002/ece3.10001

**Published:** 2023-05-02

**Authors:** Alyssa M. Willson, Hayden Gallo, Jody A. Peters, Antoinette Abeyta, Nievita Bueno Watts, Cayelan C. Carey, Tadhg N. Moore, Georgia Smies, R. Quinn Thomas, Whitney M. Woelmer, Jason S. McLachlan

**Affiliations:** ^1^ Department of Biological Sciences University of Notre Dame Notre Dame Indiana 46556 USA; ^2^ Department of Applied and Computational Mathematics and Statistics University of Notre Dame Notre Dame Indiana 46556 USA; ^3^ Mathematics, Physical and Natural Sciences Division University of New Mexico, Gallup Gallup New Mexico 87301 USA; ^4^ Indian Natural Resource Science & Engineering Program California Polytechnic State University, Humboldt Arcata California 95521 USA; ^5^ Department of Biological Sciences Virginia Polytechnic Institute and State University Blacksburg Virginia 24061 USA; ^6^ UNEP GEMS/Water Capacity Development Centre University College Cork Cork Ireland; ^7^ Division of Natural Resources Salish Kootenai College Pablo Montana 59855 USA; ^8^ Department of Forest Resources and Environmental Conservation Virginia Polytechnic Institute and State University Blacksburg Virginia 24061 USA

**Keywords:** curriculum, ecological forecasting, inclusion, STEM education, undergraduate education

## Abstract

Conducting ecological research in a way that addresses complex, real‐world problems requires a diverse, interdisciplinary and quantitatively trained ecology and environmental science workforce. This begins with equitably training students in ecology, interdisciplinary science, and quantitative skills at the undergraduate level. Understanding the current undergraduate curriculum landscape in ecology and environmental sciences allows for targeted interventions to improve equitable educational opportunities. Ecological forecasting is a sub‐discipline of ecology with roots in interdisciplinary and quantitative science. We use ecological forecasting to show how ecology and environmental science undergraduate curriculum could be evaluated and ultimately restructured to address the needs of the 21^st^ century workforce. To characterize the current state of ecological forecasting education, we compiled existing resources for teaching and learning ecological forecasting at three curriculum levels: online resources; US university courses on ecological forecasting; and US university courses on topics related to ecological forecasting. We found persistent patterns (1) in what topics are taught to US undergraduate students at each of the curriculum levels; and (2) in the accessibility of resources, in terms of course availability at higher education institutions in the United States. We developed and implemented programs to increase the accessibility and comprehensiveness of ecological forecasting undergraduate education, including initiatives to engage specifically with Native American undergraduates and online resources for learning quantitative concepts at the undergraduate level. Such steps enhance the capacity of ecological forecasting to be more inclusive to undergraduate students from diverse backgrounds and expose more students to quantitative training.

## Graphical abstract

Ecological forecasting offers the ecology and environmental science community an opportunity to reflect on how to revise the existing undergraduate ecology curriculum to meet the needs of the 21^st^ century. We capitalize on this opportunity by considering what educational resources exist and do not exist for teaching and learning ecological forecasting, as well as who has access to said resources. We present our methodology as an iterative process for curriculum revision that can be applied to the fields of ecology and environmental science more broadly.

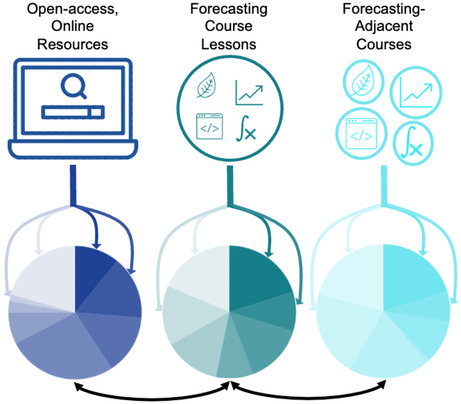

## 1 INTRODUCTION

Undergraduate ecology education prepares the next generation of scientists to address the complex environmental problems facing 21^st^ century society. Increasingly, environmental and ecological problem‐solving requires using quantitative (Barraquand et al., 2014; Farrell & Carey, 2018) and interdisciplinary (Boon & Van Baalen, 2018; NASEM, 2005) research methods and skills. Introducing undergraduate students to sub‐disciplines of ecology that focus on quantitative and interdisciplinary skills can increase students' preparedness to contribute to ecological and environmental research and problem solving in graduate school and in their careers (Farrell & Carey, 2018; Hounshell et al., 2021). Not only should education focus on training students broadly in quantitative and interdisciplinary methods, but also there is a strong need for targeted efforts that promote educational equity and inclusivity (Bowser & Cid, 2021; Graham et al., 2013). Providing opportunities to students who would not traditionally receive quantitative and interdisciplinary training enables a greater diversity of scientists to contribute a broader range of ideas and perspectives which will inherently represent more diverse interests from relevant parties (Bowser & Cid, 2021; Cheryan et al., 2017; Gardner‐Vandy et al., 2021; Graham et al., 2013; Hofstra et al., 2020; Kozlowski et al., 2022; Morrison & Steltzer, 2021; NASEM, 2021a). Numerous recent publications have highlighted the continued lack of diversity among science students and professionals (Hunter et al., 2010; Miriti, 2019, 2021; Riegle‐Crumb et al., 2019; Schell et al., 2020), particularly in quantitative and data science fields (Paxton, 2020). Consequently, we urge for greater attention to how to improve the equity of science education, as one component of addressing this pervasive problem.

We contend that ecological forecasting (EF) offers a promising approach to leveraging ecology and environmental science education for addressing 21^st^ century environmental challenges (Moore, Carey, & Thomas, 2022, Moore, Thomas, et al., 2022; Box 1). Making accurate, quantitative forecasts about the future state of ecosystems is an urgent need to improve scientific understanding of ecological phenomena and to implement appropriate policy and management decisions (Dietze, 2017a). Examples of quantitative ecological forecasts include forecasts of the global carbon cycle (Gao et al., 2011), water quality forecasts (Carey et al., 2022), and epidemiological forecasts (Oidtman et al., 2021), each of which informs policy and management. To keep pace with the growing demand for forecasts, an increasing number and diversity of scientists must be available to contribute to the discipline. This means that more scientists must be familiar with, or even have expert knowledge on, EF, including quantitative and interdisciplinary ecological research methods. Despite its importance for 21^st^ century policy and management (Bradford et al., 2020) and relevance for learning quantitative and interdisciplinary methods, EF is seldom taught at the undergraduate level.

EF not only can help address complex environmental problems, but also offers an approach to undergraduate education in which key concepts and skills, including quantitative skills and interdisciplinary team building, can be taught under a unifying framework (Box 1). We take an approach to EF consistent with the Ecological Forecasting Initiative (Dietze & Lynch, 2019), a grassroots network of interdisciplinary researchers with the collective goal of building a global community of practice around near‐term, iterative EF. This approach is grounded in the disciplines of ecology and statistics, consistent with the tradition of predictive ecology. It additionally promotes the integration of social sciences with these disciplines to improve the applicability of forecasts to end users, science communication, and science literacy, to name a few applications of social science concepts. As an emerging sub‐field with an active scholarly community, there are numerous opportunities to consider how to make EF more inclusive and equitable to students and professionals from underrepresented backgrounds. Additionally, establishing community norms in an emerging sub‐field may allow for principles of inclusivity and equity to become part of the identity of the field from its inception, an approach that may be easier to facilitate than trying to change norms of an existing discipline. For these reasons, EF, an emerging sub‐field with a suite of pedagogical benefits for undergraduate students (Box 1), offers a timely example of how to revise and expand the existing ecology and environmental sciences (EES) curriculum to better meet the needs of undergraduate students and the science workforce postgraduation. While EF is not the only way to achieve more quantitative and interdisciplinary undergraduate training, it does invite EES researchers and educators to engage in a larger conversation about the state of EES education.

BOX 1 **Ecological forecasting**

**Table:** 

*What is ecological forecasting?* Ecological forecasting (EF) is the process of producing quantitative predictions for an unknown state of an ecosystem or its services with quantified uncertainty (Carey et al., 2022; Clark et al., 2001). This sub‐field integrates theory and methods from multiple disciplines outside of ecology (Woelmer et al., 2021), including data science computer science statistics social sciences EF is a relatively new, emerging sub‐field (Dietze, 2017a; Lewis et al., 2022), meaning that opportunities for learning EF are relatively infrequent, but also that opportunities to develop curricula at the undergraduate level and to consider the equity of EF education are abundant. *Why teach and learn ecological forecasting?* Teaching undergraduate students ecology through the perspective of EF can offer instructors a way to integrate multiple pedagogical benefits in a unifying framework. Specifically, EF **Facilitates connections between scientific concepts** EF introduces students to concepts from multiple disciplines (e.g., ecology, mathematics, social sciences) under a unifying framework (i.e., EF) that can promote interdisciplinary thinking for solving complex problems (Boon & Van Baalen, 2018) and student skill building (Vogler et al., 2018) Learning about ecological modeling (a component of EF) has been shown to increase students' “systems thinking”— students' ability to recognize the interrelatedness of components of an ecological system— relative to a traditional ecology education (Carey et al., 2020) EF encourages integration of the social sciences with the natural sciences, which can increase students' perceptions of the relevance of the natural sciences to their lives (Tripp & Shortlidge, 2019) and offers students an opportunity to navigate multiple interested parties in applied science contexts (Parr et al., 2007) 2 **Improves student engagement over traditional teaching methods** EF emphasizes the real‐world applications of interdisciplinary science training (Boon & Van Baalen, 2018), which facilitates instruction using project‐based, active learning strategies that increase engagement (Graham et al., 2013; Vogler et al., 2018) Teaching EF using specific, case‐based examples (see Discussion: Current EFI education initiatives) presents the opportunity to offer students culturally relevant curriculum (Harris et al., 2020), which promotes engagement and persistence particularly among students from underrepresented backgrounds (Corneille et al., 2020; Gardner‐Vandy et al., 2021) 3 **Prepares students for the scientific workforce** Iterative EF, in which model predictions representing ecological hypotheses are repeatedly confronted with data, represents one of the most rigorous tests of ecological theory (Dietze, 2017b). Students with a background in EF enter the workforce with a suite of tools well suited to advancing scientific theory through iterative forecasts EF emphasizes quantitative skills, such as modeling, coding, and statistics, thus providing a means to introduce students to skills that are useful for students interested in pursuing careers in any domain of science (Barraquand et al., 2014) Predicting the state of ecosystems and their services under specific climate and management scenarios offers students the means to provide policymakers and interested parties with a tool for making policy and management decisions EF offers students an opportunity to become familiar with the unique benefits and challenges of contributing to interdisciplinary research, which has become increasingly in‐demand as complex global change problems require interdisciplinary solutions (NASEM, 2005, 2022) Engaging with a curriculum that includes interface with end users, science communication, and data visualization increases students' science literacy, which promotes skills such as problem solving and adaptability that are integral to the 21^st^ century workforce (Council et al., 2010)

Despite persistent calls over decades for increasing attention to be given to quantitative, predictive ecology (Clark et al., 2001; Dietze, 2017a; Houlahan et al., 2017; Jørgensen, 2002), undergraduate students in domain sciences (e.g., biology) are often not sufficiently introduced to quantitative methods to contribute to this body of research (Rawlings‐Goss et al., 2018). Many higher education institutions do not require quantitative coursework within biology and environmental science majors: Quantitative coursework may only be offered in other disciplines, without collaboration with domain science instructors (Farrell & Carey, 2018; Robeva et al., 2020). Similarly, courses on high‐level quantitative concepts, such as mechanistic modeling, are infrequently taught at the undergraduate level to ecology students. At the time of publication and to the authors' knowledge, fewer than 15 courses on EF are offered across the United States (Appendix S1, Table 1), according to both our use of self‐reported courses on EF through EFI and through a search for EF courses in the course catalogs of US land grant institutions (Appendix S1). Collectively, this results in baccalaureate graduates who may be underprepared to contribute to research addressing 21^st^ century global environmental problems, such as EF research.

In addition to relying on quantitative methods, addressing 21st century ecological research questions requires diverse teams of scientists and end users. Having diverse research teams presents multiple benefits for science and society (National Research Council, 2015). Researchers from historically underrepresented groups tend to make more novel connections between scientific concepts (Hofstra et al., 2020; Kozlowski et al., 2022; NASEM, 2021a), suggesting that more diverse research teams produce more innovative research. Increasing the diversity of scientists also means that the identities of those working on issues such as climate change better represent the identities of the people affected by the same issues (Bowser & Cid, 2021). Finally, recruiting students from historically underrepresented groups into the scientific workforce increases the number of qualified and talented workers (Cheryan et al., 2017; Graham et al., 2013). These reasons to promote diversity within scientific disciplines operate concurrently with the more important perspective that diverse research teams are morally good (Morrison & Steltzer, 2021; NASEM, 2022); the worthiness of diverse research teams should not rely on the commodification of the contributions of scientists from underrepresented backgrounds (Gardner‐Vandy et al., 2021). Making concerted efforts to improve the equity of educational opportunities represents a promising way to improve the persistence of students from diverse backgrounds into scientific disciplines (Graham et al., 2013).

Unfortunately, numerous barriers hinder the attainment of equitable science education and career opportunities in the United States. At the undergraduate level, students from marginalized ethnicities and genders show lower persistence in undergraduate science majors than White male students (Bowser & Cid, 2021). Systematic lower persistence of students from marginalized ethnicities and genders is a result of the numerous systemic barriers to participation these students face. Factors contributing to the persistence disparity include, among many other inequities, a general lack of culturally relevant science curriculum (Collins, 2018; Corneille et al., 2020; Rawlings‐Goss et al., 2018) and inequality in access to higher education resources (Dolcini et al., 2021; NASEM, 2021b; Sanders & Scanlon, 2021). Because ecology and environmental science research and curriculum has largely been constructed using a White, Western framework (David‐Chavez & Gavin, 2018), scientific topics covered in coursework, the examples used to contextualize the information, and even what scientific questions we choose to ask can be irrelevant and inaccessible to students from marginalized backgrounds (Reano, 2019).

Compounding these problems are the structural inequities in access to technology and higher education in the US. Science education can be inaccessible to students without consistent access to the internet or to computer hardware often required for homework and online learning. Students without access to these resources are disproportionately from marginalized backgrounds (Dolcini et al., 2021; Sanders & Scanlon, 2021). Similarly, highly selective higher education institutions, where instructors are more likely to have disciplinary expertise in emerging research sub‐fields such as EF, disproportionately enroll White students (NASEM, 2021b). This means students from marginalized backgrounds may not have the ability to enroll in courses on topics such as EF.

Here, we use EF to show how EES researchers and educators could begin to revise EES undergraduate education in the United States to meet the demands of the 21^st^ century scientific workforce. Because EF is an emerging sub‐field (Woelmer et al., 2021), there are few existing educational resources specific to EF, making resource development a high priority for the EF community. Consistent with recommendations from the National Academies of Sciences, Engineering, and Medicine on recruiting workers into the scientific workforce (NASEM, 2021b), we specifically emphasize developing equitable educational opportunities for undergraduate students. Leveraging the attributes of EF as an emerging, quantitative, and interdisciplinary sub‐field, we evaluate how EF can be used as one approach to updating and revising undergraduate EES curriculum in a way that simultaneously emphasizes quantitative and interdisciplinary skills and promotes educational equity.

**FIGURE 1:**
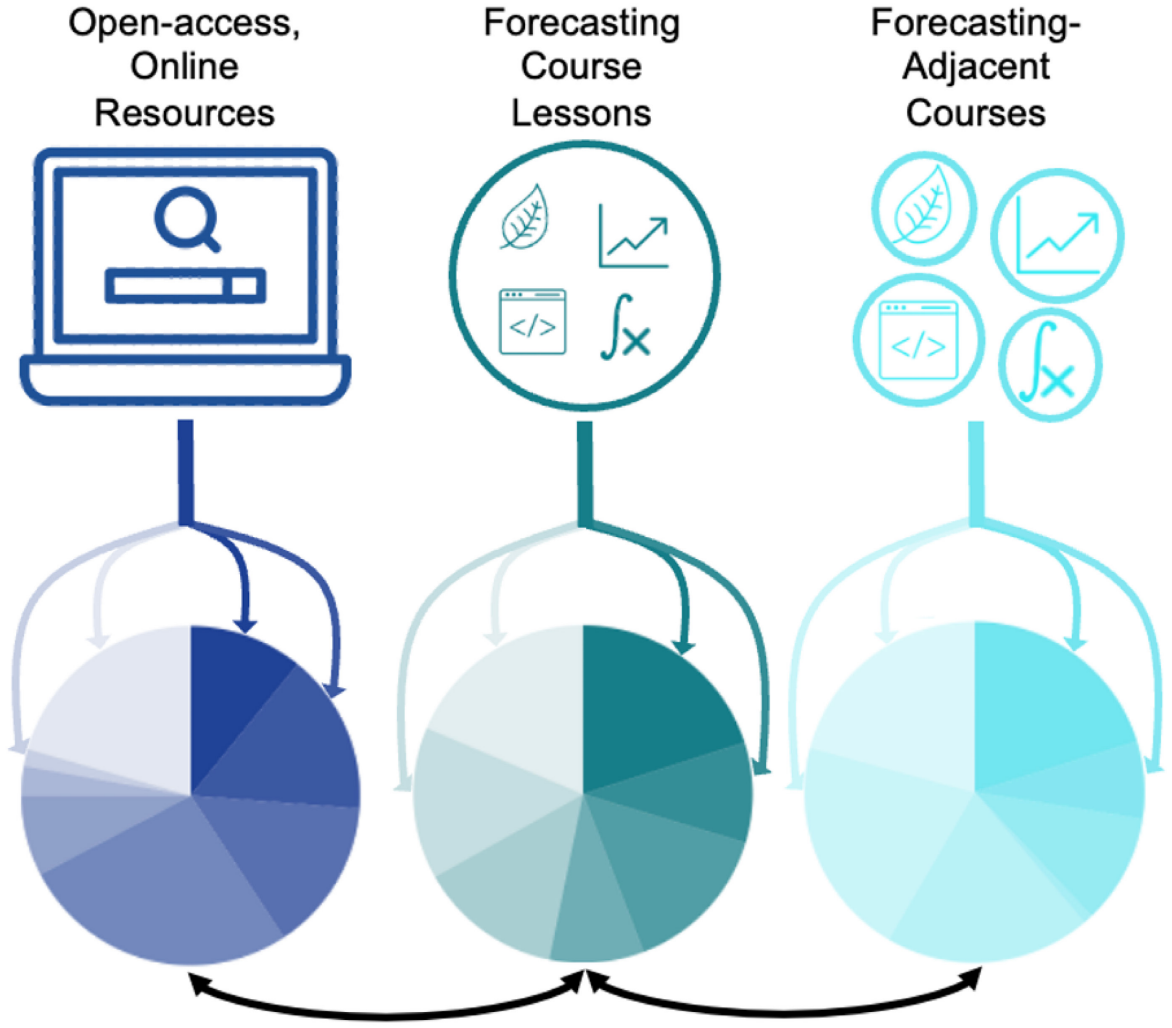
Conceptual representation of methodology. Data collection and analysis at each of our three curriculum levels. From left to right, dark blue represents the process of data collection and analysis for online resources, teal represents the process for forecasting course lessons, and light blue represents the process for forecasting‐adjacent courses. For each curriculum level, data were collected online via Google (online resources), course syllabi (forecasting course lessons), or course catalogs (forecasting‐adjacent courses). Data were organized into the forecasting topic that best represented the material covered in the resource (multi‐headed arrows) and the distribution of resources within each forecasting topic was displayed in pie charts. Black double‐headed arrows represent the comparison of the distribution of resources (ecological forecasting curriculum landscape) between forecasting course lessons and online resources (left) and forecasting course lessons and forecasting‐adjacent courses (right). The number of forecasting topics represented in the pie charts is random in this figure and serves only to show that the number of topics can differ by curriculum level.

One place to begin revising the existing EES curriculum is to understand the current curriculum landscape (NASEM, 2020, 2021a; Rawlings‐Goss et al., 2018). Understanding what online materials and courses already exist allows educators to identify gaps in the existing EES curriculum to which finite time and resources could be allocated. We first assessed the existing educational resources for gaps in the EES curriculum at three curriculum levels (Figure 1). We focus on two aspects of the EES curriculum landscape via two questions. First, we address the question “What patterns exist in the availability of resources for teaching and learning topics related to EF at the undergraduate level?” Second, we address the question “Who has access to the online resources and courses related to EF, based on who has access to the infrastructure required to access online resources and to the institutions at which courses are taught?” In response, we discuss programs the EF community has initiated to address the gaps we identified. We conclude with a discussion of future directions for the EF community, with applications to other EES sub‐disciplines.

## 2 METHODS

To examine the current state of EF undergraduate curriculum, we defined three curriculum levels (online resources; forecasting course lessons; and forecasting‐adjacent courses) (Methods: Description of Curriculum Levels). We collated resources available for teaching and learning EF at each curriculum level (Methods: Data Collection). Then, we compared the resources at each of the three curriculum levels, both in terms of how they are distributed among educational topics associated with an EF education, and how accessible they are to students from diverse backgrounds and at diverse higher education institutions.

### 2.1 Description of curriculum levels

We defined multiple curriculum levels (online resources; forecasting course lessons; and forecasting‐adjacent courses), on which we collated available resources for teaching and learning EF (see Methods: Data collection). We examine three curriculum levels to understand how many types of resources contribute to the availability and accessibility of ecological forecasting education (Figure 1).

Online resources were publicly available online, without the need to pay or ask permission to access the material. We define educational material as any course content, video, article, hands‐on learning opportunity, code, or other online material that can be used to teach or learn about EF and adjacent topics (e.g., basic statistical techniques, foundational domain knowledge) (see Appendix S1, Table 3 for a list of formats of educational material and their definitions). Our definition of online resources ignores the contributions of massive open online courses (MOOCs), a growing platform for knowledge transfer to a large quantity of students (Zawacki‐Richter et al., 2018). We chose to omit EF‐related MOOCs from our analysis of online resources to limit the complexity of our data collection and analysis procedures and we recognize that this choice impacts our characterization of online resources as generally having shallow material coverage.

### 2.2 Data collection

We collected data at each curriculum level (Figure 1). We began our search for online educational material by using Google to search terms related to each of our EF topics (defined below, “Data Categorization”) in combination with terms such as “tutorial” and “learn” (e.g., “ecology tutorial,” “learn R"). These searches were conducted iteratively from July to December 2020. We supplemented our Google searches with searches of the course and lab websites of instructors known to the authors to participate in quantitative ecology and/or ecological forecasting education (based on membership and participation in EFI and related communities [e.g., Ecological Society of America]) during the same time period. Finally, we created and distributed a Google Form through which we asked other members of the EF community to submit other known online resources, which was disseminated via EFI communications (i.e., Slack, monthly newsletter, and via word‐of‐mouth during working group meetings) during 2020 (Figure 1). A complete database of the compiled resources is available via QUBES (Willson & Peters, 2021). We acknowledge that our database of online resources is incomplete because of the great number of resources available on the internet, particularly for learning basic concepts such as statistical computing languages. However, we believe our database is a representative subsample of all resources available for learning EF.

We collated a list of forecasting courses from which we identified topics covered in individual course lessons. EFI, a grassroots organization focused on creating a community of practice around near‐term EF, houses a database of known forecasting courses on its website (https://ecoforecast.org/resources/educational‐resources/). The courses are largely self‐identified, with instructors volunteering to include their syllabus on the website; when we knew of an EF course that was not on the website, we additionally asked individual instructors to submit their syllabus for inclusion on the website. As of March 15, 2022, the database includes nine courses at the undergraduate and/or graduate educational level that fall within our definition of a forecasting course and have a syllabus available online (Appendix S1). To ensure that we did not bias our sample by including only courses from the EFI website, we searched the course catalogs (i.e., including every department) of US land grant institutions for potential EF courses and contacted the instructors of courses thought to be on EF. We chose to sample US land grant institutions because this offered a representative subsample of all US higher education institutions, with substantial representation of Minority Serving Institutions (e.g., Hispanic‐Serving Institutions, Historically Black Colleges and Universities, Tribal Colleges) and 2‐year colleges, as well as high and very high research intensity colleges and universities (Appendix S1). We found no additional courses on EF during this additional search (Appendix S1). Because we are aware of so few forecasting courses that match our criteria in the United States, we chose to include both undergraduate and graduate courses in our analysis of forecasting course lessons. For each of these nine courses, we used the syllabus to determine what topics are taught during the course. Specifically, we categorized each lesson title from the course schedule into one of the topics of EF defined below (Figure 1).

Finally, we compiled data on forecasting‐adjacent courses available at a subset of 48 higher education institutions in the United States. We randomly selected institutions from a list published by *The Edvocate* (Lynch, 2019). We chose this list because it represents a comprehensive list of all colleges and universities in the United States, 1900 higher education institutions, including 2‐year and 4‐year colleges, private and public universities, and for‐profit and not‐for‐profit institutions, and includes institutions from all 50 states. Sampling was not stratified, so our random subsample is representative of the relative frequency of different institution types (e.g., baccalaureate colleges, very high research intensity doctoral universities). For each institution, we systematically read the most recent course catalog and recorded undergraduate course names and descriptions related to EF and the forecasting topic (defined below) that best defines the content of the course. More detailed explanations about the data collection procedure for each curriculum level are available in Appendix S1.

### 2.3 Data categorization

We defined general topics that comprise EF, which we used to categorize the data we collected at each curriculum level (Figure 1). The topics represent the skills and concepts with which a student of EF should ideally become familiar during their education. We defined the forecasting topics via expert elicitation by multiple founding members of EFI, such that our topics represent EFI's current view of the requirements for a well‐rounded education in EF. The topics represent skills and concepts from multiple disciplines, including biological sciences, computer science, statistics, and social sciences (Appendix S1, Table 3). Our definition of forecasting topics using expert elicitation introduced bias in favor of the approach to EF promoted by EFI, as noted in the Introduction.

The purpose of defining EF topics was to assess the distribution of resources within broad categories of knowledge and skills students are currently learning from EF at the different curriculum levels. We classified each online resource into one or more topics that best represent the subject content. We classified each lesson from forecasting courses into one topic that best represents the material covered in the class for that lesson. Similarly, we categorized each forecasting‐adjacent course under the topic that best describes the course. Examples of our classification scheme are provided in Appendix S1, Table 3.

EF requires concepts from a suite of disciplines. Specifically, we have included three topics that may be more traditionally associated with the humanities and social sciences than the physical sciences: science communication, social sciences, and ethics. We include science communication as a foundational topic of EF because, as a field with a particular emphasis on applied research, communicating research and outcomes is an integral component of many applications of EF (Dietze, 2017b). Similarly, topics in the social sciences, including decision science and expert elicitation, offer a structured methodology for scientists interfacing with diverse interested parties and incorporating many interests into forecasting workflows, while considering ethics (e.g., data science ethics, computer science ethics, ecology ethics) ensures that applications of EF operate within ethical boundaries (e.g., ensuring that data are open source when possible, crediting knowledge from community members with authorship and acknowledgments).

Finally, we strongly believe that any ecological research should incorporate the voices and ways of knowing of all interested parties in questions of the health and preservation of our natural landscapes (David‐Chavez & Gavin, 2018; Dawson et al., 2019; Hill et al., 2020). Our forecasting topics include one type of culturally relevant education under the title “Traditional Ecological Knowledge.” We recognize that by focusing only on Traditional Ecological Knowledge, we omit other categories of culturally relevant educational material (e.g., culturally relevant educational materials for Black and Hispanic students; Corneille et al., 2020; Miriti, 2019; Rawlings‐Goss et al., 2018). However, during our data collection, we found no materials at any curriculum level that specifically focused on providing culturally relevant forecasting education to students of minority identities other than Indigenous students. Therefore, we chose to define this topic as “Traditional Ecological Knowledge” instead of “Culturally Relevant Materials” to specifically highlight the lack of materials for students from other minority ethnic/racial backgrounds.

### 2.4 Data analysis

**FIGURE 2:**
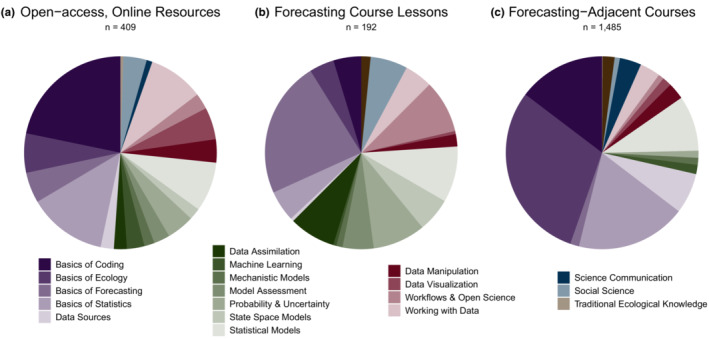
The ecological forecasting curriculum landscape differs by curriculum level. (a) Online resources. (b) Lessons in forecasting courses. (c) Forecasting‐adjacent courses. Colors are the same in all panels and correspond to the figure legend. The slices corresponding to the forecasting topics are in the same order in each panel. The colors in each panel are in the same order as in the figure legend, starting from the 12:00 position and moving counterclockwise in the plots and starting from left and moving top to bottom in the legend. Total number of resources in each curriculum level is shown below each panel's title.

Our data analysis focused on addressing two components of forecasting education attainability: (1) the distribution of resources among forecasting topics at each curriculum level (Figure 1), and (2) the accessibility of forecasting education in terms of institution type (e.g., the selectivity of the institution).

To address the distribution of resources among forecasting topics at each curriculum level (i.e., whether some topics are more represented within the available resources than others), we used $\chi^{2}$ tests using the *freqtables* package (Cannell, 2020) in the R statistical environment (R version 4.1.2) (R Core Team, 2021). We interpret the $\chi^{2}$ as indicating that resources are unevenly distributed among forecasting topics when the results are statistically significant (i.e., *p* < *α* when *α* = .05). Using a graphical representation of the data, we then specifically focused on identifying topics that are overrepresented and underrepresented at each curriculum level. For EF curriculum development, the overrepresentation of some topics relative to others suggests that there are sufficient resources available for those topics and additional resource development could focus on other topics. On the other hand, underrepresentation of some topics relative to others highlights gaps to fill with further resource development.

As a second approach to characterizing the availability of resources among curriculum levels, we used Fisher's exact tests to quantify the dissimilarity between different curriculum levels. Specifically, we compared the distribution of resources among forecasting topics between online resources and forecasting course lessons, and between forecasting‐adjacent courses and forecasting course lessons. We compared forecasting course lessons to the other two curriculum levels because forecasting course lessons comprise our best representation of what EF experts believe students should learn to become ecological forecasters. Fisher's exact test was chosen because this test is similar to a chi‐squared test when the dimensions of the contingency matrix are greater than 2 × 2 but is robust to zero frequencies. Fisher's exact tests were computed using the *freqtables* package (Cannell, 2020), altering the default function to use the hybrid approximation option for large contingency tables and 2000 Monte Carlo permutations. In this way, we not only considered the distribution of resources within each curriculum level, but also relative to the current standard (i.e., forecasting course lessons) within the sub‐field.

We considered the distribution of course‐based resources at different curriculum levels among institution types. We assigned each institution a type (e.g., R1 = doctoral universities with very high research activity, B = baccalaureate universities) using Carnegie Classifications, as defined by Indiana University's Carnegie Classification of Institutions of Higher Education database (https://carnegieclassifications.iu.edu/index.php). We then grouped the Carnegie Classifications to simplify the interpretation of institution type (see Appendix S1, Table 4 for the grouping system of the nine classification types).

We conducted a logistic regression using the glm() function in the *stats* package (R Core Team, 2021) to quantify the difference in forecasting‐adjacent course offerings by institution type. Our logistic regression used a binary variable corresponding to institution type A/B versus M/D. The binary classification A/B (n_A/B_ = 535) corresponds to the following institution types: Associate's Colleges (A), Associate's/Bachelor's Colleges (A/B), Bachelor's Colleges (B), and Tribal Colleges and Universities (TC). The binary classification M/D (n_M/D_ = 950) corresponds to institution types Master's Colleges and Universities–Smaller Programs (M3), Master's Colleges and Universities–Larger Programs (M1), Doctoral Universities–High research activity (R2), Doctoral Universities–Highest research activity (R1), and Doctoral/Professional Universities (D/PU). The A/B and M/D categories are the response variable in the logistic regression and the forecasting topics into which courses fell are the predictor variables. We used this binary categorization to increase the statistical power of the analysis by including more courses in fewer institution type categories. We grouped Tribal colleges (TC) with associate's and baccalaureate institutions (A/B) because the two Tribal colleges included in this analysis do not offer degrees higher than bachelor's degrees. We removed the topics “Model Assessment” and “Traditional Ecological Knowledge” from this analysis because only one course was available for each topic, which limited our ability to make inference regarding these topics. We interpret the coefficients of the model as the odds of an institution being an associate's or baccalaureate institution (A/B) relative to a master's or doctoral institution (M/D), given the frequency of offering courses in a given forecasting topic.

## 3 RESULTS

### 3.1 The EF curriculum landscape differs by curriculum level

Our quantitative analysis of resources for teaching and learning EF reveals differences in how resources are distributed among forecasting topics, with different gaps existing at different curriculum levels (Figure 2). The distribution of resources within the forecasting topics is uneven at each curriculum level (online resources: $\chi^{2}$ = 364, df = 18, *n* = 409, *p* < .005; forecasting course lessons: $\chi^{2}$ = 163, df = 17, *n* = 192, *p* < .005; forecasting‐adjacent courses: $\chi^{2}$ = 2891, df = 17, *n* = 1485, *p* < .005), indicating that some topics are more intensively covered than others at each curriculum level. Further, the distribution of forecasting course lessons among the forecasting topics is more even than the distribution of online resources (Fisher's exact test: n_online_ = 409, n_lessons_ = 192, *p* < .005) and forecasting‐adjacent courses (Fisher's exact test: n_courses_ = 1485, n_lessons_ = 192, *p* < .005). This implies that many topics are over‐ and underrepresented within online resources and forecasting‐adjacent courses with respect to developing a comprehensive EF curriculum at each of these levels, as defined by evenly covering the topics identified via expert elicitation to comprise an EF education. For example, a disproportionately high number of online resources are dedicated to basic concepts of computer coding (Figure 2) relative to both the number of resources dedicated to other topics among online resources and the number of resources dedicated to basic concepts of computer coding at the other curriculum levels (Table 1). On the other hand, basic concepts of forecasting, such as what a forecast entails and why prediction is important in the sciences, are an underrepresented topic among both online resources and forecasting‐adjacent courses relative to its representation among forecasting course lessons (Figure 2, Table 1).

**TABLE 1:** Examples of over‐ and underrepresented topics at each of the three curriculum levels, based on Figure 2. These examples serve to reinforce the examples we use in the Discussion. Readers are invited to identify other over‐ and underrepresented topics based on the data and Figure 2.

Curriculum level	Example topics overrepresented	Example topics underrepresented
Online resources	Basics of coding	Basics of Forecasting
		Ethics
Forecasting course lessons		Ethics
		Science communication
		Traditional ecological knowledge
Forecasting‐adjacent courses	Basics of ecology	Basics of forecasting
		Data assimilation
		State space models

It is also worth noting that some topics are not represented at all at one or more curriculum levels. Ethical considerations in forecasting and in related disciplines (ecology, or computer and data sciences) were not represented in our database of online resources and only three of 192 forecasting course lessons in three of nine courses covers forecasting ethics. This topic highlights important considerations when conducting EF research, including who should be involved in deciding what forecasts are made and whether data should be made publicly available. The forecasting topics (see Appendix S1, Table 3), Science Communication and Traditional Ecological Knowledge are also not represented in forecasting courses. Among our sample of forecasting‐adjacent courses, there are no courses specifically covering Data Assimilation or State Space Models. These topics that are not represented in a curriculum level represent particularly persistent gaps in EF curricula (Table 1).

### 3.2 Availability of forecasting and forecasting‐adjacent courses differs by institution type

**FIGURE 3:**
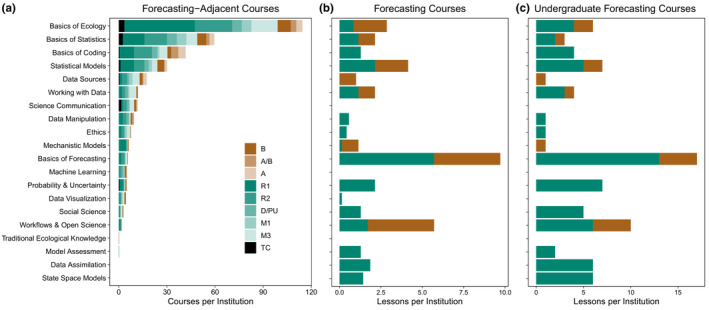
Institution type influences the availability of resources in each forecasting topic. Institution type is depicted by a simplification of Carnegie Classification system. Categories in the legend are as follows: A = Associate's college, A/B = Associate's/Bachelor's College, B = Bachelor's College, D/PU = Doctoral/Professional University, M1 = Master's Colleges and Universities – Larger Program, M3 = Master's Colleges and Universities – Smaller Program, R1 = Doctoral University – Very High Research Activity, R2 = Doctoral University – High Research Activity, TC = Tribal College. See Appendix S1, Table 4 for the list of institutions per institution type and the Carnegie Classification system. (a) The number of forecasting‐adjacent courses per institution. Courses per institution were computed by dividing the total number of courses per forecasting topic and institution type by the number of institutions in each institution type. (b) Graduate and undergraduate forecasting course lessons per institution. Lessons per institution were computed by dividing the total number of lessons per forecasting topic and institution type by the number of institutions in each institution type. (c) Undergraduate forecasting course lessons per institution. As in (b), but only visualizing undergraduate forecasting courses and excluding forecasting courses at the graduate level.

We next considered whether there are patterns in the availability of resources at the course‐based curriculum levels (i.e., forecasting course lessons and forecasting‐adjacent courses) across institution types. In general, more forecasting and forecasting‐adjacent courses are available at doctoral universities than at colleges and universities offering only associate's, bachelor's, or master's degrees (Figure 3; Table 2). Forecasting courses are currently almost exclusively taught at doctoral universities with very high research activity (institution type R1, *n* = 7/9), while only one forecasting course is taught at a highly selective baccalaureate institution (institution type B, Smith College; Figure 3b,c) and one is taught outside of the university setting (EFI summer course).

**TABLE 2:** Coefficients from the logistic regression comparing the prevalence of courses in each forecasting topic among associate's‐ and bachelor's‐granting institutions (A/B) and master's‐ and doctoral‐granting institutions (M/D).

Forecasting topic	Estimate [95% CI]	*p*‐value
Intercept	1.06 [0.81, 1.38]	.68
Basics of ecology	2.24 [1.60, 3.16]	<.001
Basics of forecasting	9.46 [2.10, 42.7]	.003
Basics of statistics	1.74 [1.20, 2.51]	.003
Data manipulation	1.89 [0.93, 3.84]	.07
Data sources	0.89 [0.55, 1.44]	.63
Data visualization	1.47 [0.60, 3.61]	.39
Ethics	8.52 [2.45, 29.6]	<.001
Machine learning	2.68 [1.00, 7.20]	.05
Mechanistic models	3.08 [0.95, 9.96]	.06
Probability & Uncertainty	1.73 [0.61, 4.96]	.29
Science communication	1.38 [0.74, 2.55]	.30
Social science	1.51 [0.47, 4.89]	.48
Statistical models	1.43 [0.92, 2.22]	.11
Workflows & Open science	0.38 [0.11, 1.27]	.11
Working with data	3.44 [1.65, 7.16]	<.001

*Note*: The left column includes the names of the forecasting topics used to predict the institution type at which the course is offered (either associate's/baccalaureate institution or master's/doctoral institution). The middle column includes the coefficient estimates and 95% confidence intervals (CIs) in brackets. The right column includes the *p*‐values of the coefficients, indicating whether the coefficient is significantly different from zero. Rows are shaded gray if the *p*‐value is significant at α = .05. The *p*‐value for the topic “Machine Learning” is below α before rounding. Coefficients have been exponentiated to represent odds.

There is more heterogeneity in the type of institutions offering forecasting‐adjacent courses than in the institutions offering forecasting courses (Figure 3a). While more forecasting‐adjacent courses are offered at master's and doctoral universities (Figure 3a), other institution types (i.e., offering lower degrees) offer courses corresponding to almost every forecasting topic (Appendix S1, Table 3). Specifically, associate's colleges offer a range of courses relevant to forecasting, particularly within topics associated with computer and data sciences (Figure 3a). Our logistic regression model offers insight into the topics that forecasting‐adjacent courses cover at different types of institutions. Specifically, we interpret the coefficients of our logistic regression (Table 2) as the odds of an institution being an associate's‐ or bachelor's‐granting institution (A/B) relative to a master's‐ or doctorate‐granting institution (M/D), given the frequency of offering courses of a given forecasting topic. Using this interpretation, three of the six topics indicating a higher odds of an institution being A/B (i.e., offering less advanced degrees) are introductory topics, while three are related to more advanced topics (Table 2). No topics representing advanced quantitative concepts except Machine Learning significantly contribute to the difference between associate's and baccalaureate institutions and master's and doctoral institutions, although two topics (Data Manipulation and Mechanistic Models) are marginally nonsignificant.

## 4 DISCUSSION AND RECOMMENDATIONS FOR NEXT STEPS

### 4.1 Gaps are visible in the existing resources at each curriculum level

Previous literature has discussed the importance of understanding the current curriculum landscape as a first step in improving curriculum in higher education (NASEM, 2020, 2021a; Rawlings‐Goss et al., 2018). We identified resources that already exist and highlighted gaps where finite resources could be allocated to build a more comprehensive curriculum in the context of EF. For example, we show that there is a need to introduce high‐level quantitative skills to undergraduate students in forecasting and forecasting‐adjacent courses (Figure 2, Appendix S1). This finding is consistent with previous research indicating that certain data science skills are underrepresented in undergraduate education (Emery et al., 2021) and that undergraduate EES curriculum is an appropriate venue for introducing students to high‐level quantitative skills (Barraquand et al., 2014; Carey et al., 2020). This finding also underscores previous research findings that students recognize their own lack of quantitative training in undergraduate ecology curriculum and retroactively desire more training in quantitative concepts and skills (Barraquand et al., 2014). These skills additionally have the potential to improve students' problem‐solving abilities and scientific workforce preparedness (Barraquand et al., 2014; Farrell & Carey, 2018; Hounshell et al., 2021).

Introductory concepts are particularly overrepresented among online resources and forecasting‐adjacent courses. This is relevant because these resources can supplement formal instruction on EF, offering educational opportunities to students without access to forecasting courses. The overrepresentation of introductory concepts is likely because these topics are applicable to many science domains and are not specific to EF. This is encouraging because such introductory concepts can similarly contribute to other quantitative, interdisciplinary EES curricula that are not specifically related to EF. Further, the overrepresentation of some topics points to a positive feature of the EF curriculum landscape: multiple resources are likely to exist on the same introductory concepts, offering different perspectives and ways of teaching information to students with diverse learning styles. We suggest that further resource development efforts may not need to focus on introductory concepts and instead can focus on underrepresented advanced topics.

The complete absence of certain forecasting topics helps identify gaps where new resources could be developed to offer a more comprehensive suite of resources to approach EF in the way promoted by EFI. For example, online resources could be developed to allow students to explore the ethical implications of making ecological forecasts. Similarly, topics related to disseminating forecasts to interested parties could be incorporated as specific lessons into more forecasting courses or could be offered as forecasting‐adjacent courses at more institutions. Finally, the fact that there are few resources for incorporating non‐Western ways of knowing into EF courses highlights the need for more diverse instructors teaching EF courses and contributing to resource and course development. We recognize that EES educators and researchers will make their own decisions about which topics warrant their own courses or course lessons. For example, the topic of state space models may be more suited for incorporation into existing statistical modeling courses, rather than having a course exclusively on this topic. We anticipate that further refinement of our list of topics as more EF educators contribute to our research will help the EF community reach consensus on what topics deserve their own courses and course lessons. Similarly, other EES sub‐disciplines should consider what topics are appropriate for entire courses and course lessons upon applying our approach.

### 4.2 Limitations to gap identification

Educating undergraduate students is a complex undertaking, meaning that our results should be considered in the context of how students learn. For example, educators understand that some topics can be more suitable to one curriculum level than another. The inclusion of ethics as a lesson within forecasting courses may help students make more connections between ethics and the development of forecasts (Emery et al., 2021; NASEM, 2020, 2021b). Additionally, teaching interdisciplinary topics such as ethics and science communication within forecasting courses is consistent with science instructors' role and virtue responsibilities (i.e., the responsibilities conferred upon science instructors as science instructors and as moral agents in the practice of teaching science students, respectively) to teach students these topics within the context of the scientific disciplines (Sethy, 2018). When topics such as ethics are embedded into disciplinary courses, our method for determining the topics taught within forecasting‐adjacent courses may have failed to identify that ethics was taught. Therefore, it is possible that we have underestimated how often ethics and similar topics are taught. Similarly, the mode of knowledge transmission, whether online (e.g., online resources or virtual courses) or in‐person (e.g., traditional courses) may impact student comprehension and learning (Adedoyin & Soykan, 2020). While we did not consider explicitly the mode of knowledge transfer in this study, we encourage future studies to do so. Finally, it is possible that we mischaracterized the availability of resources by failing to account for the depth to which a topic is covered in a given resource. For example, if many resources cover statistical models at a surface level, students may in reality have fewer resources available to them to learn statistical models than to learn a topic that is covered by few resources, but more in depth.

Similar to considering the context in which forecasting topics are taught, by assuming that forecasting course lessons represent the “ideal” distribution of resources among forecasting topics, we recognize that we introduce some bias into our analysis. Specifically, the composition of forecasting courses is heavily dependent upon who is teaching forecasting and which resources the instructors are using to structure their lessons. Similarly, many current EF instructors share resources, such as textbooks and syllabi (e.g., on EFI's website), contributing to strong nonindependence between EF courses in our database. Owing to both the nonindependence between EF courses and common demographics between students in EF courses (e.g., mainly graduate students in ecology and environmental sciences, often with prior exposure to quantitative concepts and coding and attending wealthy universities), there may be shared expectations between EF courses regarding the concepts and skills students have prior to the course. For example, many current EF courses expect students to enter EF courses with foundational knowledge in ecology and statistics. We, therefore, advocate for repeating our approach to analyzing the EF curriculum landscape and continuing to use forecasting course lessons as the expected distribution of courses, as EF matures as a discipline.

### 4.3 Patterns exist in the accessibility of resources at each curriculum level

Equally important to the distribution of existing resources among forecasting topics is the accessibility of resources. Accessibility of online resources is restricted to students with reliable broadband internet access and personal computers. Significant portions of students in rural areas, Tribal communities, low‐income households, and from traditionally underrepresented racial and ethnic backgrounds are less likely to have reliable internet and computer access than “traditional” students, who are mainly from urban and suburban, middle class, White households (Dolcini et al., 2021; Sanders & Scanlon, 2021).

Aside from online resources, the accessibility of courses, both forecasting and forecasting‐adjacent, is biased toward highly selective institutions. Based on our representative database of forecasting and forecasting‐adjacent courses, we show that all forecasting courses and a substantial portion of forecasting‐adjacent courses are taught at doctoral‐granting institutions with very high research activity (R1 or highly selective baccalaureate institutions (B; Figure 3, Table 2). We recognize that we may have missed some forecasting courses, especially if they are not labeled as such in course descriptions. Similarly, by analyzing a subsample of all US higher education institutions, we have missed a great deal of forecasting‐adjacent courses at unsampled institutions and even in sampled institutions, some courses without sufficient course description online were inevitably missed. Nevertheless, our analysis highlights that students living in areas with limited options for higher education (e.g., areas with only community college options; “academic‐match education deserts”) may be unable to enroll in forecasting courses (Klasik et al., 2018). This disparity is compounded by the fact that 80% of White Americans enroll in the top 500 higher education institutions, where forecasting courses are more likely to be offered, while 75% of students from underrepresented racial and ethnic backgrounds do *not* enroll in the top 500 institutions (NASEM, 2021b). This highlights the fact that the racial/ethnic identity and socioeconomic status of students influence the accessibility of forecasting education, disparities which should become a high‐priority consideration in the development of EF curriculum.

Although we did not consider them in this analysis, the rise of MOOCs offers a promising way to deliver course content on topics related to ecological forecasting, including quantitative concepts, to students with access to any higher education institution, not just those with access to highly selective institutions (Zawacki‐Richter et al., 2018). However, learning via MOOCs presupposes that students have access to the internet and the computer hardware required for attending the course and completing assignments. As is true for any online resource, these assumptions do not hold for many students living in rural communities, Tribal communities, low‐income households, among other living arrangements (Dolcini et al., 2021; Sanders & Scanlon, 2021). We advocate for future iterations of this analysis to include MOOCs and for the continued development of MOOCs related to ecological forecasting and quantitative ecology. We additionally urge resource developers, educators, and researchers to additionally consider the needs of students in such low‐internet and resource environments.

The fact that most advanced topics related to EF (e.g., mechanistic and statistical models, probability and uncertainty) are equally represented at associate's and bachelor's‐granting institutions compared to master's and doctoral‐granting institutions (Table 2) indicates that advanced quantitative curriculum is similarly developed at both of these institution types. Students at both institution types could benefit from more training in advanced quantitative concepts, consistent with the fact that advanced quantitative concepts tend to be less well represented among forecasting‐adjacent courses than introductory concepts (Figure 2). Meanwhile, the fact that fewer forecasting‐adjacent introductory courses are available across our subsample of master's and doctorate‐granting institutions highlights that these could benefit from coordination of introductory coursework with partnering 2‐year institutions (e.g., community college to university programs).

### 4.4 Application to other disciplines

The analysis of both the availability and accessibility of undergraduate educational resources should not be interpreted as limited to EF. Researchers and educators in other sub‐disciplines of EES can assess the availability of resources for teaching and learning their discipline by identifying the topics relevant to the discipline. Further, two of our main conclusions are applicable to any EES researcher or educator interested in improving the availability and accessibility of EES education. First, the relative lack of educational resources in more advanced statistical and computational concepts suggests that additional quantitative resources could improve the education of EES undergraduate students. Second, the fact that EF resources at all three curriculum levels have strong barriers to access that disproportionately affect students from marginalized communities is applicable to the entire EES community. Increasing the accessibility of educational resources across curriculum levels should be a priority for EES educators and researchers as a community.

### 4.5 Progress and future directions

#### 4.5.1 Current education initiatives

Here, we describe several initiatives we have implemented to begin addressing identified gaps in EF education. These initiatives were co‐developed by the co‐authors while the analysis of the availability and accessibility of ecological forecasting education was undertaken. The initiatives are designed to meet two objectives: (1) increase representation of underrepresented minority students in data science education in the United States and (2) increase the number of resources for teaching and learning about high‐level quantitative concepts related to ecological forecasting. To increase the representation of underrepresented minority students in data science, we have developed a partnership between the Ecological Forecasting Initiative and staff and faculty from three Minority Serving Institutions with high Native American enrollment (Salish Kootenai College, University of New Mexico—Gallup, and California Polytechnic State University—Humboldt). The objective of this partnership is to identify the specific barriers to participation of students from marginalized backgrounds and attending under‐resourced higher education institutions and begin reducing the identified barriers. To increase the resources available for teaching and learning quantitative concepts, members of the Ecological Forecasting Initiative partnered with Environmental Data‐Driven Inquiry and Exploration (EDDIE) to develop educational modules on quantitative concepts. Importantly, these modules emphasize specific components of ecological forecasting that we identified as being underrepresented, including uncertainty quantification and data assimilation. Through these current initiatives, we are beginning to address some of the gaps in both the availability and accessibility of ecological forecasting education identified in our analysis.

Native American undergraduate students are a subset of underrepresented minority students in the sciences in the United States, with pervasive socioeconomic barriers to success in the university context (Alexiades et al., 2021). Consistent with objective (1), we are currently evaluating existing data science and EF resources to address the accessibility of EF education for Native American undergraduate students. It is understood that lack of internet access is a barrier to many students across the country; however, this problem is exacerbated among Native American communities due to chronic disenfranchisement of Tribal citizens and the continued legacy of colonization within the United States. According to the FCC, across the country, about 6% of individuals lack internet access (Federal Communications Commission, 2012). In contrast, 18% of Tribal reservation residents do not have access to the internet. Of the limited access available on reservations, there is a high reliance on smartphones and cellular services for access to the internet. As a result, we are evaluating whether (1) materials can be used in low to no internet environments; (2) materials can be used on mobile devices, the only computers some students have; and (3) materials can be modified to meet technical needs of communities, such as being modified to be used in no internet environments or on smartphones. This initiative has started with considering the needs of students at the University of New Mexico—Gallup, a 2‐year institution with high enrollment of members of the Navajo Nation.

Bridging the gap between objectives (1) and (2), increasing representation of underrepresented minority students and increasing resources for teaching and learning quantitative concepts, we have integrated Traditional Ecological Knowledge into an R Data Analysis course at Salish Kootenai College (SKC). SKC is a Tribal college serving members of nearly 70 Tribes, making efforts to teach students how to incorporate the questions, issues, and experiences of their communities into applied science (e.g., via ecological forecasting) particularly relevant. Students were taught to use the R statistical computing language to quantitatively analyze water quality data using a K'avi Tribe Water Quality Dataset. Water quality data analysis is relevant to the production of mandatory reports that many Tribal governments must send to the US Environmental Protection Agency (EPA) annually. The K'avi Tribe, the Water Quality Dataset, and the reference watershed are fictional, created so that no one Tribe would feel singled out or excluded when this class is taught at SKC. The complexity of water resources within the fictional watershed and the 10‐year dataset reflect realistic landscape and water quality conditions present at many Reservations in the Intermountain West, US. Indeed, the dataset is an amalgam of data collected by one co‐author (Smies) for five different Tribes in Montana, US.

Additionally, the Traditional Ecological Knowledge (TEK) sites included in the K'avi Tribe Water Quality Dataset mirror the spiritual and cultural uses of water resources by many Tribes in the West (Rattling Leaf Sr, 2022). Today, the EPA (the primary funder of Tribal water quality programs) only evaluates monitoring data based upon physical and biological parameters. Tribes are left to create their own TEK analysis methods and many Tribal managers are not equipped with the resources to do so. As a result, TEK values are not given equal protection via Tribal water quality standards, despite their importance. In response, students in the SKC R Data Analysis course were tasked with ranking TEK values associated with the important spiritual and cultural sites in the K'avi Tribe watershed. Then, the students were taught coding strategies that allowed them to combine the quantitative and qualitative datasets. Data visualizations of the combined data created new and more powerful ways to evaluate the physical and cultural health of the watershed. By ascribing equal value to both the Western and Indigenous scientific approaches, students' analyses more fully described ecosystem function. This approach encourages students to learn quantitative concepts within a framework that facilitates making connections between coursework and their lives and cultural values, an approach that has been shown to improve the persistence of Native American students in undergraduate studies (McMahon et al., 2019).

We address objective (2) by expanding the breadth of resources available for teaching and learning ecological forecasting via Macrosystems EDDIE. Macrosystems EDDIE modules provide students and faculty with hands‐on, real‐world learning activities to bolster their understanding of macrosystems ecology through modeling and forecasting (MacrosystemsEDDIE.org). The newest four modules (modules 5–8) are primarily focused on teaching macrosystems ecology and EF concepts using National Ecological Observatory Network data (Carey et al., 2020; Farrell & Carey, 2018; Hounshell et al., 2021; Moore, Thomas, et al., 2022, Moore, Carey, & Thomas, 2022; Woelmer et al., 2022). Each of the four forecasting‐focused Macrosystems EDDIE modules covers different components of the iterative forecasting cycle, which include building a model, quantifying uncertainty, generating a forecast, communicating a forecast, assessing a forecast, and updating a forecast with observations.

#### 4.5.2 Future directions

Coursework, such as forecasting and forecasting‐adjacent courses, cannot be fully inclusive without considering in‐classroom inclusive best practices (Rawlings‐Goss et al., 2018), such as active learning strategies (Corwin et al., 2018; Graham et al., 2013), including culturally relevant examples (Harris et al., 2020), and providing alternative evaluation methods (Miriti, 2019), among others. Our analysis neglected this critical component of EF curriculum by focusing on the courses themselves, rather than the context in which they are taught. Future efforts should include more opportunities for instructors to learn about inclusive pedagogy best practices, opportunities for instructors and forecasting practitioners to discuss barriers to inclusive instruction, and the implementation of working groups aimed toward addressing the identified barriers.

Numerous recent publications have highlighted the necessity of targeted interventions to improve the persistence of students from traditionally underrepresented racial and ethnic groups, including Black, Hispanic, and Indigenous students, in scientific disciplines (Bowser & Cid, 2021; Cheryan et al., 2017). We have undertaken numerous efforts to develop curricula that address the needs of Indigenous students. However, few efforts within the EFI community have focused on the needs of other racial and ethnic minorities, including Black and Hispanic students. Strategies to improve Black and Hispanic student persistence can be similar to those targeting Indigenous students, including the incorporation of Black students' cultural values into the curriculum (Collins, 2018; Corneille et al., 2020), addressing local community problems through coursework (Corneille et al., 2020), and applying the curriculum to students' lived experiences (Harris et al., 2020). Future efforts should focus on identifying and implementing strategies for developing EF curriculum that improves Black and Hispanic student persistence, including partnerships with Black and Hispanic educators and forecasting practitioners. Additionally, future EF curriculum development efforts should consider providing resources and courses in languages other than English.

## AUTHOR CONTRIBUTIONS

**Alyssa M. Willson:** Conceptualization (lead); data curation (lead); formal analysis (equal); investigation (lead); methodology (lead); validation (lead); visualization (equal); writing – original draft (lead); writing – review and editing (lead). **Hayden Gallo:** Data curation (supporting); formal analysis (equal); investigation (supporting); methodology (supporting); visualization (equal); writing – review and editing (supporting). **Jody A. Peters:** Methodology (supporting); project administration (lead); supervision (equal); writing – review and editing (equal). **Antoinette Abeyta:** Writing – original draft (supporting). **Nievita Bueno Watts:** Conceptualization (supporting); writing – review and editing (supporting). **Cayelan C. Carey:** Funding acquisition (equal); writing – original draft (supporting); writing – review and editing (equal). **Tadhg N. Moore:** Writing – original draft (supporting). **Georgia Smies:** Writing – original draft (supporting); writing – review and editing (supporting). **R. Quinn Thomas:** Funding acquisition (equal); writing – original draft (supporting); writing – review and editing (equal). **Whitney M. Woelmer:** Writing – original draft (supporting); writing – review and editing (equal). **Jason S. McLachlan:** Funding acquisition (equal); supervision (equal); writing – review and editing (equal).

## FUNDING INFORMATION

This work was supported by the NSF Research Coordination Network grant 1926388, and an Alfred P. Sloan Foundation grant. A.M.W. was supported by an NSF Graduate Research Fellowship and an Arthur J. Schmitt Leadership Fellowship in Science and Engineering from the University of Notre Dame. C.C.C., T.N.M., R.Q.T., and W.M.W. were supported by a grant for Macrosystems EDDIE (DEB‐1926050).

## CONFLICT OF INTEREST STATEMENT

The authors declare no competing interests.

### OPEN RESEARCH BADGES

This article has earned an Open Data badge for making publicly available the digitally‐shareable data necessary to reproduce the reported results. The data is available at https://doi.org/10.6084/m9.figshare.c.5995435.v1.

## Supporting information


Appendix S1.

## Data Availability

All data presented in this study are available via the Figshare data repository (https://doi.org/10.6084/m9.figshare.c.5995435.v1).
