# Supplementary material for: Assessing opportunities and inequities in undergraduate ecological forecasting education
Source: Ecol Evol. 2023 May 2;13(5):e10001. doi: 10.1002/ece3.10001 (PMC10154799; doi:10.1002/ece3.10001)
Supplement: Supplementary file 1 — Appendix S1. [file ECE3-13-e10001-s001.docx]

**Appendix 1: Course Data Collection**

We collected data on the availability of resources teaching different topics related to ecological forecasting (EF) at three curriculum levels: (1) online resources, (2) forecasting courses lessons, and (3) forecasting adjacent courses. Here, we describe in more detail the data collection procedures for forecasting course lessons and forecasting adjacent courses. In addition, we provide tables describing the Carnegie classification of the institutions with EF courses (Appendix Table 1), the formats of online resources collected (Appendix Table 2), definitions of the forecasting topics (Appendix Table 3), and a list of the higher education institutions used to identify forecasting-adjacent courses along with their Carnegie classifications (Appendix Table 4).

*Forecasting Course Lessons*

Forecasting courses were identified using a published table of EF courses from the Ecological Forecasting Initiative’s (EFI’s) website (<https://ecoforecast.org/resources/educational-resources/>). The course list is up-to-date as of March 18, 2022, although some forecasting courses have not been included on the website for several reasons. First, EFI may be unaware of some EF courses by instructors not integrally involved in EFI. Second, EFI has been unable to obtain course information and/or syllabi from some courses. However, the courses available on EFI’s website are many of the first EF courses offered in the U.S., making them a reliable foundation by which to analyze the state of EF education at its beginning.

Of the 17 forecasting courses available on the EFI website as of March 18, 2022, we excluded eight courses. First, we excluded the syllabus titled “Ecological Forecasting and Informatics 2019” taught by Michael Dietze because an updated syllabus from the same course is available under “Ecological Forecasting and Informatics 2021” also taught by Michael Dietze. Second, we excluded the courses titled “ESPM-288: Reproducible & Collaborative Data Science” taught by Carl Boettiger and “Ecological Forecasting Seminar” taught by Brett Melbourne because these courses were taught in a highly unstructured format centered around student projects, making data collection on each course lesson impossible. Third, we excluded the course “An Overview of Structured Decision Making - ALC3183 - Resources” taught by Michael Runge and Sarah Converse because this course is specifically on structured decision making, which is only one component of EF and not representative of a full EF education. Fourth, we excluded the course “Dinámica Ecológica y Predicciones (Ecological Dynamics and Predictions)” taught by Peter Adler and Juan Manuel Morales because this is the same course as another one on the list, but taught in Spanish. Finally, we excluded the courses “Quantitative Ecology and Evolution,” “Machine Learning for Ecology,” and “Data Science for Biological Research” taught by Brett Melbourne because these courses are not specifically on EF, but cover environmental data science more broadly. This left us with a total of nine EF courses.

For each of the nine EF courses, we categorized each course lesson into one of the forecasting topics we defined. Course lessons were defined as specific units of instruction that corresponded to each course period or week (depending on the structure of the course). An example syllabus is presented in Appendix Table 5. Some dates were excluded. Introductory lessons (e.g., “Introduction”) and concluding lessons (e.g., “Concluding remarks”) were excluded if there was no associated text or assignment that would indicate that the lesson included teaching related to EF. Any dates that indicated that there was no class (e.g., “Independent work time,” “Class canceled,” “Break”) were excluded. Any catch-up lessons (e.g., “Answering questions,” “Ask me anything”) were excluded.

Some courses required modifications to data collection specific to that course. In the case of the course titled “Topics in Statistical and Data Sciences - Ecological Forecasting,” the nine course periods related to course projects were combined into three lessons, one for each of the groups: “Parameter Estimation,” “Time Series Forecasting,” and “Wildfire Biomass Estimation” because each group presented three updates. For “Ecological Dynamics and Forecasting,” each bullet titled “Discussion,” “Lecture,” or “Lab” was considered its own lesson because this was the finest resolution for which we could assign a forecasting topic. For “Topics in Ecological Forecasting,” the topic of each lesson was determined from the chapter of the course’s associated textbook that was to be read for each class period. Finally, lessons for the course titled “NEFI 2020 Short Course” were determined according to the blocks of time on the calendar (e.g., blocks of one- to two-hour intervals) instead of by calendar date because the course is only one week in duration. Additionally, only lectures were included for this short course.

For each forecasting course, we recorded the URL to the syllabus, the course name, the instructor’s name, the institution at which the course was taught, the education level at which the course was offered (i.e., undergraduate, graduate, or mixed undergraduate/graduate) based on the institution’s course numbering system and the course description, and the Carnegie Classification of the institution.

Due to the small sample size of EF courses, our data includes a mixture of graduate, undergraduate, and mixed graduate-undergraduate courses. We consider the impact of including both graduate and undergraduate courses in the analysis by including separate visualizations for all courses and only undergraduate courses.

*EF Course Search*

We conducted an additional search for potential EF courses within the course catalogs of U.S. land grant institutions in order to ensure that our method of collecting forecasting course syllabi from EFI’s website did not bias our results. We focused on land grant institutions because these institutions are a subset of all higher education institutions in the U.S. and include institutions of multiple Carnegie classifications (e.g., including high research intensity doctoral universities, community colleges, Tribal colleges). A complete list of land grant institutions can be found via the National Institute of Food and Agriculture’s website (<https://www.nifa.usda.gov/about-nifa/how-we-work/partnerships/land-grant-colleges-universities>). We searched each of the institutions listed on the National Institute of Food and Agriculture’s website (<https://www.nifa.usda.gov/about-nifa/how-we-work/partnerships/land-grant-colleges-universities>), including all campuses in the University of California and University of Hawaii systems, except for D-Q University, which lost accreditation in 2005.

For each institution, we searched the most recent course catalog for the term “forecast*” among course titles and descriptions. We then evaluated each instance of the term “forecast*” for its applicability to EF. We excluded courses related to business/financial forecasting, weather forecasting, hydrological forecasting, and statistical forecasting, as determined by course descriptions, because these courses did not relate to EF.

We contacted the instructors of courses that we interpreted as potentially teaching EF to request a recent syllabus for these courses. In total, we identified fourteen potential EF courses among 129 land grant institutions. Among the fourteen potential EF courses, four courses were excluded because the instructor(s) did not respond to our request, three courses were excluded because the instructor(s) declined to provide a syllabus, two courses were determined to be on the topic of hydrologic forecasting, one course was determined to be on the topic of economic/price forecasting, one course was considered not on EF by the instructor, and two were determined to not emphasize forecasting by inspecting the course syllabi (one on climate science and one on ecological modeling). We concluded that we could reasonably assume that the EF courses available on EFI’s website represent the breadth of EF courses available at the time of analysis in the U.S.

*Forecasting Adjacent Courses*

Data on forecasting-adjacent courses were collected by looking at the course catalogs of a random sample of higher education institutions in the U.S. Institutions were identified using a published list from *The Edvocate* of all higher education institutions in the U.S., including four-year public and private institutions, two-year institutions (e.g., community colleges, technical colleges), Tribal colleges, and for-profit institutions. We selected random institutions by vectorizing the list of institutions from *The Edvocate* and using a random number generator in the statistical program R to select entries in the vector object.

A small number of institutions were excluded. Some institutions (Carnegie Science, South Central Career Center, Fortis Institute, University of Tennessee Health Science Center, Notre Dame de Namur University) were excluded because they do not offer degrees at the undergraduate level (i.e., associate’s, bachelor’s) and/or do not offer undergraduate degrees that are applicable to EF. Two institutions randomly selected from our list had closed (Concordia College New York, Concordia University Portland, Oregon) and one institution had merged with another institution (Austin Graduate School of Theology) since the publication of the list of higher education institutions in 2018. One institution (California Institute of Integral Studies) only offered options to finish undergraduate-level degrees, rather than offering an entire degree program. Our final random sample included 48 higher education institutions.

For each institution, we located the most recent course catalog on the institution’s website. We then read the course name and description for every undergraduate course offered. We identified forecasting-adjacent courses by determining whether a course was related to one of the forecasting topics. For example, a course titled “Data Visualization” can be categorized under the forecasting topic by the same name. For more examples, see Appendix Table 3. Each course applicable to EF was only categorized into one forecasting topic, even if multiple topics might be applicable. If more than one forecasting topic was applicable to a given course, the topic that appeared most central to the mission of the course (e.g., by course name, emphasis on course description) was chosen. For how specific courses were classified, we refer the reader to the data available on [Figshare](https://figshare.com/collections/Assessing_opportunities_and_inequities_in_undergraduate_ecological_forecasting_education/5995435/1).

Some courses were systematically excluded from our dataset. Introductory biology courses (e.g., “General Biology”) without a specific emphasis on ecology were excluded because we considered them more basic than what is necessary for an EF education. Similarly, biology (e.g., “Botany,” “Zoology”) and forestry (e.g., “Forest Resource Management”) courses not specific to ecology were excluded from the “Basics of Ecology” topic. Calculus courses more advanced than Calculus I were excluded because they are more advanced than what is required for an EF education. Finally, courses offered only at the high school or graduate level and courses offered for zero credits were excluded.

| **Carnegie Classification** | **Type** | **Institutions Included** |
| --- | --- | --- |
| Baccalaureate Colleges: Arts & Sciences Focus | B | Smith College |
| R1: Doctoral Universities – Very High Research Activity | R1 | Boston University |
|  |  | University of California, Berkeley |
|  |  | University of California, Santa Cruz |
|  |  | University of Colorado, Boulder |
|  |  | University of Florida |
|  |  | University of Notre Dame |
|  |  | Utah State University |
|  |  | Virginia Tech* |
| NA | NA | Ecological Forecasting Initiative Summer Course |

Appendix Table 1: Carnegie Classifications for, simplified institution types for, and portion of the analysis in which each of the eleven forecasting courses was used. The left column contains Carnegie Classifications from Indiana University’s database, the second column includes our simplified institution type, the third column includes each institution hosting an ecological forecasting course, and the final column includes the portion of the analysis in which each course was used. Note that two courses did not have sufficiently detailed syllabi to be used to analyze the distribution of lessons among forecasting topics (Portion of Analysis: Geographic only) and one course was taught outside of the university setting and thus was not included in the geographic analysis (Portion of Analysis: Forecasting topic only). All other courses were used in both parts of the analysis (Portion of Analysis: Forecasting topic; Geographic). * denotes multiple courses taught at the same institution.

| **Material Type** | **Definition** |
| --- | --- |
| Article | Online short texts |
| Course Material | Archived material from past synchronous or asynchronous academic course |
| External Repository | Links to other collections of resources (e.g., QUBES) |
| Module | Short, hands-on lessons |
| NetLogo Lab | Module dependent on NetLogo software |
| Textbook | Full online textbooks |
| Video | Recorded lectures, video tutorials, etc. |
| Workshop | Recurring synchronous learning opportunity |

Appendix Table 2: Types of online resources included in our database.

| **Forecasting Topic** | **Definition** | **Example** |
| --- | --- | --- |
| Basics of Coding | Introduction to programming languages used in quantitative ecology | Forecasting-adjacent course titled “Programming in R” |
| Basics of Ecology | Introduction to basic ecological concepts | Forecasting-adjacent course titled “Ecology and the Environment” |
| Basics of Forecasting | Introduction to the utility and general structure of ecological forecasts | Forecasting course lesson titled “The Iterative Forecasting Cycle” |
| Basics of Statistics | Introduction to statistical methods | Online video titled “State Space Models” |
| Data Assimilation | Introduction to techniques for formally combining data with model predictions | Online video titled “Introduction to Particle Filters” |
| Data Manipulation | Resources for learning how to format, manipulate, and process data for analysis | Forecasting course lesson titled “Tidy Data with Tidyverse” |
| Data Sources | Resources for learning how to acquire data used in ecological forecasting | Forecasting course lesson titled “Acquiring Satellite Data” |
| Data Visualization | Resources for making figures and graphics to represent data and forecasts | Online video titled “Plotting Trendlines with ggplot” |
| Ethics | Introduction to ethical considerations related to ecological forecasting, data science, and related disciplines | Forecasting course lessons titled “Guidelines for Deciding When to Publish Your Data” |
| Machine Learning | Introduction to machine learning techniques for ecological forecasting | Forecasting-adjacent course titled “Introduction to Machine Learning” |
| Mechanistic Models | Introduction to developing and interpreting process-driven models | Forecasting-adjacent course titled “Introduction to Earth System Modeling” |
| Model Assessment | Introduction to techniques for assessing mechanistic and statistical model performance | Online article titled “Interpretation of Akaike and Bayesian Information Criteria” |
| Probability & Uncertainty | Resources for working with and interpreting probability-based uncertainty | Forecasting course lesson titled “Uncertainty Partitioning” |
| Science Communication | Resources for learning how to communicate science to audiences within and outside of the academy (e.g., policymakers) | Forecasting-adjacent course titled “Science Writing” |
| Social Science | Introduction to social science concepts related to either expert elicitation or making informed policy and management decisions using ecological forecasts | Forecasting-adjacent course titled “Structured Decision Making” |
| State Space Models | Introduction to the specific class of statistical models of state space models | Forecasting course lesson titled “Using State Space Models” |
| Traditional Ecological Knowledge | Introduction to the incorporation of Traditional Ecological Knowledge with Western scientific knowledge | Online module titled “Native Case Studies in Conservation” |
| Workflows & Open Science | Resources for learning how to make reproducible workflows | Online module titled “Basic Github Commands” |
| Statistical Models | Introduction to developing and interpreting statistical models | Forecasting-adjacent course titled “Applied Linear Models” |
| Working with Data | Resources with hands-on practice working with actual data sets | Online module titled “Analyzing Eddy Covariance Data” |

Appendix Table 3: Forecasting topics and their definitions. All online resources, forecasting course lessons, and forecasting-adjacent courses were classified into forecasting topics. A theoretical example of each topic is provided in the right column.

| **Carnegie Classification** | **Type** | **Institutions Included** |
| --- | --- | --- |
| Associate’s Colleges: High Career & Technical – High Nontraditional | A | Chippewa Valley Technical College  Gateway Technical College  Neosho County Community College  Walla Walla Community College |
| Associate’s Colleges: Mixed Transfer/Career & Technical – High Nontraditional |  | Garden City Community College  Mid-Plains Community College |
| Associate’s Colleges: Mixed Transfer/Career & Technical – High Traditional |  | Copper Mountain College  Delta College  Guilford Technical Community College |
| Associate’s Colleges: High Career & Technical – High Traditional |  | Del Mar College  Lamar State College – Port Arthur |
| Associate’s Colleges: High Transfer – Mixed Traditional/Nontraditional |  | Illinois Central College |
| Associate’s Colleges: High Transfer – High Traditional |  | Diablo Valley College  Mineral Area College |
| Associate’s Colleges: Mixed Transfer/Career & Technical – Mixed Traditional/Nontraditional |  | City College of San Francisco  San Bernardino Valley College |
| Associate’s Colleges: High Transfer – High Nontraditional |  | Central Oregon Community College |
| Associate’s – Public Urban-serving Single Campus |  | Cedar Valley College |
| Baccalaureate/Associate’s Colleges: Associate’s Dominant | A/B | Midland College |
| Baccalaureate/Associate’s Colleges: Mixed Baccalaureate/Associate’s |  | Vermont Technical College |
| Baccalaureate Colleges: Diverse Fields | B | Culver-Stockton College  Goshen College  University of Arkansas at Pine Bluff |
| Baccalaureate Colleges: Arts & Sciences Focus |  | Centenary College of Louisiana  Swarthmore College  Vassar College |
| M3: Master’s Colleges and Universities – Smaller Programs | M3 | Eastern Connecticut State University  University of Tennessee Martin  University of Wisconsin Stevens Point |
| M1: Master’s Colleges and Universities – Larger Programs | M1 | Clarion University  Fitchburg State University  Lynn University  Nyack College  Saint Francis University |
| D/PU: Doctoral/Professional Universities | D/PU | Alliant International University  Fresno State  University of Charleston  University of Saint Joseph Connecticut  University of Texas at Tyler |
| R2: Doctoral Universities – High Research Activity | R2 | Eastern Michigan University  New Mexico State University  Rochester Institute of Technology  Thomas Jefferson University  University of Alaska Fairbanks |
| R1: Doctoral Universities – Very High Research Activity | R1 | Auburn University  University of Illinois Urbana-Champaign |
| Tribal Colleges | TC | Blackfeet Community College  United Tribes Technical College |

Appendix Table 4: Carnegie Classifications and simplified institution types for each of the 48 higher education institutions used in our analysis of forecasting-adjacent courses. The left column contains Carnegie Classifications from Indiana University’s database, the middle column groups the Carnegie Classifications into a simplified system including only nine institution types, and the right column contains each institution included in our analysis.

| Day | Topic | Activity | Reading (O = optional) |
| --- | --- | --- | --- |
| 1/25 | Intro/Syllabus | Lecture | Preface |
| 1/27 | Overview | Lecture | Chapter 1 |
| 1/29 | The command prompt is my friend | Hands-on | 1. R Primer  O: Google R tutorials  O: Git/Github tutorials |
| 2/1 | From models to forecasts | Lecture | Chapter 2  (Currie 2019) |
| 2/3 | Logistic Growth | Hands-on | 2. From Models to Forecasts |
| 2/5 | Dynamic Models | Lecture | TBA |
| 2/8 | Data, large and small | Lecture | Chapter 3  O: Data One Best Practices |
| 2/10 | Open Science | Discussion | (Powers and Hampton 2019) |
| 2/12 | Tools for big data | Hands-on | 3. Big Data |
| 2/16 Tues | Informatics of Model-Data Fusion | Lecture | Chapter 4  Team Science: (Read et al. 2016) |
| 2/17 | Workflows | Hands-on | Pair Coding |
| 2/19 | Forecast Ethics | Discussion | (Hobday et al. 2019) |
| 2/22 | Intro to Bayes | Lecture | Chapter 5  O: (Ellison 2004)  O: Bayesian Regression |
| 2/24 | BUGS/JAGS | Hands-on | JAGS Primer |
| 2/26 | Expert Elicitation | Discussion | (Morgan 2014) |
| 3/1 | Characterizing Uncertainty | Lecture | Chapter 6  O: Fitting Uncertainties  O: Hierarchical Bayes |
| 3/3 | State-space models | Lecture | Chapter 6 |
| 3/5 | State-space models | Hands-on | Chapter 8  6. State Space |
| 3/8 | Invasive species | Discussion | (Ibanez et al. 2014) |
| 3/10 | Fusing data sources | Lecture | Chapter 9 |
| 3/12 | Data-Fusion | Hands-on | 8. Tree Rings  O: (Clark et al. 2007) |
| 3/15 | Biodiversity | Discussion | Chapter 7 |
| 3/17 | Propagating Uncertainty | Lecture | Chapter 11 |
| 3/19 | Uncertainty Partitioning | Hands-on | Uncertainty Partitioning  O: (Dietze 2017) |
|  |  |  |  |
| 3/22 | Carbon | Discussion | Chapter 12 |
| 3/24 | Data Assimilation: Analytical | Lecture | Chapter 13  O: (Wikle and Berliner 2007) |
| 3/26 | Kalman Filter | Hands-on | 9. Kalman Filter |
| 3/29 | Data Assimilation: Monte Carlo | Lecture | Chapter 14  O: (Evensen 2009) |
| 3/31 | BU Wellness Day | <no class> | <no class> |
| 4/2 | Ensemble KF | Hands-on | 10. Particle Filter |
| 4/5 | Infectious disease | Discussion | Chapter 15 |
| 4/7 | Assessing Model Performance | Lecture | Chapter 16 |
| 4/9 | Model Assessment | Hands-on | 11. Model Assessment |
| 4/12 | Teamwork SWOT | Discussion | No reading |
| 4/14 | Scenarios & Decision Support | Lecture | Chapter 17 |
| 4/16 | Stakeholder exercise | Hands-on | Activity |
| 4/19 | NO CLASS |  | Patriot’s Day |
| 4/21 | Decision Support | Discussion | (Memarzadeh and Boettiger 2019, Miller et al. 2019) |
| 4/23 | Final projects | Hands-on |  |
| 4/26 | Final projects | Hands-on |  |
| 4/28 | Final Thoughts | Lecture | Chapter 18  (Dietze et al. 2018) |
| 5/4 9-11 | FINAL | Presentation |  |

Appendix Table 5. Example syllabus for a forecasting course. The syllabus is from the course titled “Ecological Forecasting and Informatics—Spring 2021” taught by Michael Dietze at Boston University. Forecasting course lessons were defined as each row of the table, corresponding to the dates that the class meets. The lessons were categorized into one of the forecasting topics using the “Topic” column. Topics can be repeated for multiple lessons. Some lessons were removed from the analysis, as explained in “Forecasting Course Lessons.”
